# The Issue of Burnout and Work Satisfaction in Younger GPs—A Cluster Analysis Utilizing the HaMEdSi Study

**DOI:** 10.3390/ijerph15102190

**Published:** 2018-10-08

**Authors:** Oliver Hirsch, Charles Christian Adarkwah

**Affiliations:** 1 Department of Psychology, FOM University of Applied Sciences, 57078 Siegen, Germany; oliver.hirsch@fom.de; 2 Department of Health Services Research and General Practice, Faculty of Life Sciences, University of Siegen, 57076 Siegen, Germany; 3 Department of General Practice and Family Medicine, Philipps-University, 35043 Marburg, Germany; 4 CAPHRI School for Public Health and Primary Care, Department of Health Services Research, Maastricht University, 6229 GT Maastricht, The Netherlands

**Keywords:** primary health care, general practitioners, burnout, job satisfaction, cluster analysis

## Abstract

The shortage of general practitioners (GPs) in Germany has become a relevant problem. Therefore, it is important to find the determinants that make primary care more attractive, and which support GPs remaining in practice. Our aim in this exploratory study was to search for relevant GP subgroups and their characteristics in order to find starting points for improvements or interventions. We attempted a comprehensive survey of all GPs in the German region of Siegen-Wittgenstein with about 280,000 inhabitants. There were 158 GPs in the total population; 85 of these (53.8%) took part in the study. There were 64 male GPs (75.3%) in our sample. The mean age of the participants was 53.5 years (SD 8.93). The questionnaire was composed of demographic questions, questions regarding future perspectives, the Motivation for Medical Education Questionnaire (MoME-Q), the Maslach Burnout Inventory (MBI), and the Work Satisfaction Questionnaire. K-means cluster analyses were used for subgrouping. A 2-cluster solution had good statistical quality criteria. Cluster 1 was characterised by elderly GPs who more frequently had a resident physician in their practices. These GPs had low burnout scores and high work satisfaction scores. Cluster 2 consisted of younger GPs who less frequently had a resident in their practices. They had average burnout scores according to published norms and lower work satisfaction scores. There seems to be an age cohort effect regarding burnout and work satisfaction. Having a resident physician seems to be protective. Interventions should be designed for younger GPs, especially members of generation Y, to reduce burnout and improve work satisfaction.

## 1. Introduction

Half of all general practitioners (GPs) in Germany are not satisfied with their working conditions [1,2]. Low work satisfaction, meaning the amount of pleasure one feels doing one’s job, with high stress levels and an unsatisfactory work–life balance can lead to symptoms of burnout [3,4]. Job stress and burnout are closely related to work satisfaction. In turn, work satisfaction is determined by a number of factors. These can be classified into job resources (e.g., skill discretion, relations with colleagues) and job demands (e.g., workload, conflicts at work) [5]. In Germany, the GP training lasts five years. For three years, residents are trained in hospitals, whereafter during the final two years the training takes place in GP practices before residents get permission to go for board examination. European studies show that GPs in rural areas seem to be more affected by burnout symptoms than are GPs in urban regions [3,6]. In Siegen-Wittgenstein, the HaMEdSi study (Hausaerzte (GPs) for Medical Education in Siegen-Wittgenstein) was carried out in order to investigate the motivation for medical education, and GPs’ work satisfaction and risk of burnout. The district of Siegen-Wittgenstein represents a typical and representative rural region in Germany. In this region, medical students will be educated and trained in the near future, as a new medical campus is being established in cooperation with an existing medical school located at another city, about 90 km away. Furthermore, GPs, at least in the German setting, will be challenged by the Masterplan Medical Education 2020. With this Masterplan, the importance of “General Practice” will significantly increase. General Practice will become a major subject within the medical education curriculum [7].

On the one hand, every student will have to complete three months of General Practice education within the sixth study year (practical year); on the other hand, General Practice will become a mandatory examination subject in the final oral examination (3rd part of the examination). This matter of fact is of high relevance as a large number of GP practices for teaching and training will be necessary in order to comply with this demand. There is a shortage of doctors, especially general practitioners (GPs), in the rural district of Siegen-Wittgenstein, and this will increase dramatically in the near future. This may well add to the above-mentioned problem of work satisfaction and risk of burnout. Parts of the study have already been published, where good statistical quality criteria and good model fits in confirmatory factor analyses could be shown for both instruments used, the Maslach Burnout Inventory (MBI) and the Work Satisfaction Questionnaire [8,9,10]. 

Cluster analysis has proven to be a useful technique for research and practice in primary care. It is beneficial to base a study on an analytical approach, as demonstrated by the study to subgroup low-back-pain patients by cluster analysis [11]. Important decisions regarding treatment and resource allocation can be based on the results of such analyses. Therefore, our aim was to subgroup GPs based on their characteristics in the HaMEdSi study in order to search for structural improvements and to design future interventions for GPs in the area of stress reduction and to enhance work satisfaction. Furthermore, a k-means cluster analysis was able to differentiate between the different communication styles of Dutch and US GPs, finding that US GPs focused more on biomedical topics while Dutch GPs had higher proportions of socioemotional counseling [12]. In another instance, cluster analysis was used to examine the profiles of GPs who are involved in the management of mental health care. The study revealed that GPs were primarily involved in the treatment of patients with common mental disorders but less in the treatment of patients with serious mental disorders [13]. French GPs could be divided into four clusters regarding their attitudes toward treating terminally ill patients. Important conclusions for practical palliative care can be drawn from such analyses [14]. Another study was performed in the context of alcohol treatment in primary care. GPs could be divided into two subgroups based on their attitudes toward patients with alcohol problems. Alcohol-specific treatment programs might be tailored to address these specific attitudes in order to enhance effectivity [15]. These examples demonstrate that cluster analysis can be a useful technique in primary care research. Accordingly, we applied this technique in our study to subgroup GPs regarding personal and practice characteristics in order to draw conclusions for the provision of primary care in Germany in the future.

## 2. Materials and Methods 

### 2.1. Study Design

We conducted a study in which all general practitioners in the district of Siegen-Wittgenstein were invited to take part. The contact details of GPs were obtained through the Association of Statutory Health Insurance Physicians (KVWL). GPs were asked about their work satisfaction and risk of burnout. Furthermore, they were asked about their motivation to participate in the medical education of students, as well as their work perspective. In this paper, we focus on subgrouping GPs based on their ratings in the study.

This survey was performed in general practices in the area of Siegen-Wittgenstein in Germany between October 2017 and January 2018. All GPs in the area were sent a written invitation with a detailed study description, an informed consent form and the study questionnaire. After four weeks, all GPs who had not responded received a telephone reminder from a member of the study team. An invitation to participate was also sent by email to all members of the local doctor’s association, in which most of the GPs hold a membership. In January 2018, recruitment was terminated.

The study was performed in accordance with the Declaration of Helsinki and approved by the research ethics committee of the University of Marburg (Az.: Studie 127/17). We have obtained informed consent from each study participant.

### 2.2. Instruments

We designed a questionnaire which contained items about the current job situation and future perspectives. This includes questions about their own career path as well as future job satisfaction and retirement plans. We further developed the Motivation for Medical Education Questionnaire (MoME-Q), which includes the factors commitment and personal benefit; these emerged as significant in confirmatory factor analyses. Cronbach-α and omega-coefficients of “commitment” with 16 items were around .90, while the factor “personal benefit” had 8 items with Cronbach-α and omega coefficients being around .80 [16].

The Work Satisfaction Questionnaire is comprised of 17 items to be scored on a 7-point scale from “1—very dissatisfied” to “7—very satisfied” [9,17]. The questionnaire is based on the main components of work satisfaction identified by prior qualitative research conducted by the Society of General Internal Medicine Career Satisfaction Study Group [18]. The items address satisfaction with relationships with patients, peers, nurses and other nonmedical staff, time for family, friends or leisure, workload and work stress, administrative burden, autonomy in treating patients, autonomy in referring patients to a specialist, intellectual stimulation at work, opportunities for continuing medical education, enjoyment of work, respect and prestige, type of payment mechanism, current income, overall quality of care, and job satisfaction in general. The Work Satisfaction Questionnaire has a five-factor structure, including patient care (4 items, Cronbach-α = 0.76), burden (4 items, α = 0.79), income-prestige (3 items, α = 0.83), personal rewards (3 items, α = 0.71), and professional relations (2 items, α = 0.66). Furthermore, a global item asks for the respondent’s satisfaction with their current job situation. This item correlates with the subscale scores from 0.39–0.71 [12]. The instrument was shown to be sensitive to structural changes in healthcare systems [19]. In our study population we were able to confirm the five-factor structure by a confirmatory factor analysis. The reliability coefficients in our sample were as follows: The Cronbach-α coefficient of the patient care subscale was 0.78; the omega coefficient was 0.79. The Cronbach-α coefficient of the burden subscale was 0.76; the omega coefficient was 0.77. The Cronbach-α coefficient of the income-prestige subscale was 0.58; the omega coefficient was 0.62. The Cronbach-α coefficient of the personal rewards subscale was 0.70; the omega coefficient was 0.71. The Cronbach-α coefficient of the professional relations subscale was 0.65, and the omega coefficient was 0.65 [8].

We used the German version of the Maslach Burnout Inventory (MBI) to assess occupational burnout. The MBI is designed to measure an enduring state of experienced burnout, which is borne out by the stability of its scores over time [10]. The MBI comprises 22 items which the respondents score on a 7-point scale from “0—never” to “7—every day”. It consists of 3 subscales, namely “emotional exhaustion” (9 items), which measures exhaustion at work, “depersonalization/lack of empathy” (5 items), which measures loss of empathy and emotional distance to others, and “personal accomplishment” (8 items), which measures competence and positive attitude towards work. The three-factor structure was confirmed [20], the Cronbach-α of the emotional exhaustion scale was 0.85, of the personal accomplishment subscale 0.71, and of the depersonalization subscale just 0.48. Other studies found higher internal consistencies for this subscale with Cronbach-alphas of 0.69 and 0.86, respectively [21,22]. Convergent and discriminant validity of the MBI could be demonstrated [21,22]. In our study population we were able to confirm the three-factor structure by a confirmatory factor analysis. The reliability coefficients in our sample were as follows: the Cronbach-α coefficient of the emotional exhaustion subscale was 0.84, the omega coefficient was 0.85. The Cronbach-α coefficient of the depersonalization/loss of empathy subscale was 0.69, the omega coefficient was 0.71. The Cronbach-α coefficient of the personal accomplishment subscale was 0.70, the omega coefficient was 0.71 [8]. All values can be classified as satisfactory to high.

### 2.3. Statistical Methods

There were a maximum of 5 missing values on single items of the MBI and the Work Satisfaction Questionnaire. These were replaced by the k-nearest-neighbour algorithm (kNN) [23] using R version 3.3.2 [24] package Visualization and Imputaion of Missing Values (VIM) [25]. We performed k-means cluster analyses generalised to all scales of measurement with squared Euclidean distances [26]. The k-means procedure identifies relatively homogeneous subgroups while maximizing the variability between clusters. Variables with mixed scaling can be handled in cluster analysis [26,27]. Calculations were done with ALMO 15 [28], which includes a k-means algorithm able to handle the different scaling of our variables and the large sample size. This program provides statistical measures for evaluating the appropriateness of a cluster solution (F value, η^2^). The F value is calculated following analysis of variance. It can be regarded as the maximum F value, as the variation between clusters is maximized [27,29]. This Fmax value does not follow an F distribution, in contrast to analysis of variance. Therefore, no test of significance is possible. η^2^ represents the effect size in a general linear model (GLM). It is an omnibus effect size when examining the cluster solution as a whole, and a partial η^2^ when examining the contribution of single variables to the cluster solution. Only relevant variables should be included in a cluster analysis. Irrelevant variables can destroy the clustering and prevent an interpretable solution from appearing [29,30]. Consequently, cluster analysis is an iterative process. Therefore, we ran several analyses and excluded variables with no meaningful contribution. Variables with an η^2^ < 0.04, which signal a small contribution to cluster formation, were excluded from further analyses. We compared the resulting clusters on classification variables with effect sizes Cramér V (0.20 to 0.39 corresponding to a moderate association, ≥0.40 signaling a strong association) [31] and Cohen´s d (0.2 showing a small effect, 0.5 a medium effect, and 0.8 a large effect) [32].

## 3. Results

### 3.1. Study Population

There are a total of 158 GPs in the district of Siegen-Wittgenstein taking care of a population of about 283,000 inhabitants. Of these, 85 (53.8%) took part in the study and completed the questionnaire without receiving any kind of reimbursment. This participation rate was comparable to other studies in primary care [33]. There are 64 male GPs (75.3%) in our sample. The gender distribution conforms to the proportions in the population in this specific area. The mean age of the participants is 53.5 years (SD 8.93) with a median of 54 years, a minimum age of 32 and a maximum age of 73 years. The majority (91.8%) are practice owners, work full-time (90.6%) and work in a group practice (67.1%). The average study participant has worked in private practice for 18.41 years (mean, SD 9.8) with a range of between 2 and 43 years. Most of them are specialized in general practice (51.8%), whereas 24.7% specialized in internal medicine; 20.0% have both specialisations. The minority (3.5%) are “Praktischer Arzt” (medical practitioner) without any further specialisation. This denomination has been disestablished and was taken out of the regulation for further education in 1992. It is notable that despite the higher proportion of bureaucracy and increasing number of patients, 94% of our respondents would become GPs again. Further characteristics of the study participants have already been published elsewhere [8] and can be found in Table 1.

### 3.2. Cluster Analysis

After the first cluster analysis, the following variables were excluded as they did not contribute significantly to cluster formation: gender (η^2^ = 0.02), actually involved in the qualification of future GPs (η^2^ = 0.002), existence of practice after retirement (η^2^ = 0.003), single/group practice (η^2^ = 0.001), Motivation for Medical Education Questionnaire (MoME-Q) [16] scale commitment (η^2^ = 0.037), MoME-Q personal benefit (η^2^ = 0.011).

A 2-cluster solution was best interpretable and resulted in an F value of 22.15 and an η^2^ of 0.211, meaning that 21.1% of the variance can be explained by this partitioning. Consequently, this cluster solution possesses good quality criteria [26,30,34]. Cluster 1 comprises 43 GPs (50.6%), Cluster 2 consists of 42 GPs (49.4%). Table 2 displays the contribution of each variable to cluster formation, given by effect size η^2^, and Table 3 shows descriptive statistics of the classification variables and the respective effect sizes of the differences between the clusters.

The highest contributions to cluster formation have MBI scale scores for “depersonalization” and “exhaustion”, and Work Satisfaction Questionnaire scale scores for “patient care” and “personal rewards” (Table 2).

Clusters 1 and 2 differ in several respects. In Cluster 1, a higher proportion of practices had a resident physician. Physicians in Cluster 1 are older and have been practicing longer than those in Cluster 2. The effect sizes signal medium effects. Physicians in Cluster 1 complain less about exhaustion and about depersonalisation/lack of empathy, and they are more satisfied with patient care, more satisfied regarding personal rewards, and more satisfied with professional relations. The effect sizes for these differences are all large. They have a higher sense of personal accomplishment, are more satisfied regarding burden, and more satisfied with income and prestige. These are medium effects.

## 4. Discussion

### 4.1. Main Findings

Two distinct clusters emerged from our analyses. The highest contributions to cluster formation had MBI scale scores for “depersonalization” and “exhaustion”, and Work Satisfaction Questionnaire scale scores for “patient care” and “personal rewards”. In Cluster 1, GPs were older, had practised longer, more frequently had a resident physician, and they had low burnout and high work satisfaction scores. The opposite pattern occurred in Cluster 2, which was characterised by younger GPs.

### 4.2. Interpretation in Relation to Existing Literature

We found a clear age effect regarding burnout and work satisfaction in GPs. This finding is supported by a Spanish study [35]. Here, work satisfaction for GPs was positively associated with years of experience. Furthermore, a Swiss study by Bovier and Perneger [17] showed that older physicians have the highest scores regarding work satisfaction, whereas physicians in training (mostly young physicians) are least satisfied. In contrast to our study, the latter paper did not focus on general practitioners and also included doctors working in clinics. Studies in different areas also report similar results. In German service employees, age was also negatively correlated with symptoms of burnout, and positive associations were shown for professional efficacy and engagement. More effective emotion regulation strategies were hypothesized to be a decisive factor in older employees [36].

Our results might reveal a first cohort effect with younger GPs being less resistant to occupational stress. These are important results for future generations, especially for those belonging to generation Y (born between 1981 and 2000). Members of generation Y set high priorities on education, freedom, equality, and family life [37]. They prefer to work independently and express their ideas [38].

The GPs in Cluster 1 had low burnout symptoms according to norms published by Soler et al. [39], while GPs in Cluster 2 had an average burnout symptom load. The work satisfaction of GPs in Cluster 1 was higher than in other published studies, although these refer to Swiss GPs [9]. Keeping GPs in practice is important for the German health system, as there is already a remarkable shortage of GPs in rural areas. Therefore, tailored interventions should be designed for GPs in Cluster 2, to enable them to reduce burnout symptoms and to improve work satisfaction. As a consequence, the probability that this subgroup will remain in practice longer might be higher.

### 4.3. Strengths and Limitations

The sample size could have been larger, but we attempted a total population survey in a specific region, and the participation rate was comparable with other studies on primary care [32]. Nevertheless, our results should be replicated with independent and larger samples, as cluster analysis is an exploratory method. GPs were the target group of our study and our conclusions should therefore be restricted to this specific group and cannot be generalised to other specialties.

## 5. Conclusions

A multivariable technique like cluster analysis enables the efficient subgrouping of GPs. The results of cluster analyses can help in the design of special interventions for distinct subgroups of GPs to improve their work satisfaction and to reduce symptoms of burnout. This can be an important contribution to ensuring that GPs who are in danger of being negatively affected by such variables are empowered to remain in practice. This, in long-term consideration, can help to make it more attractive for resident physicians to become GPs, and can therefore also be seen as one approach to reduce the lack of GPs in rural areas. 

## Figures and Tables

**Table 1:** Demographic characteristics of study participants (*n* = 85).

**General Characterictics**	
Gender	75% male 25% female
Practice ownership	92% practice owners 8% practice employees
Specialization	52% General Practice 25% Internal Medicine 20% General Practice and Internal Medicine 3% none
Practice size	33% single practice 67% group practice
Modus of work	91% full-time 9% part-time
Would become GP again	94% yes 6% no
**Teaching Experience**	
Cooperation with medical school/status of an adacemic teaching practice	14% yes 86% no
Any preexisting teaching experience	59% yes 41% no
One-day observation	45%yes 55% no
Two-week rotation	17% yes 83% no
Clinical elective	57% yes 43% no
Practical Year	11% yes 89% no
Lectures at a university	3% yes 97% no
Visited didactics training within last 2 years	6% yes 94% no
**Perspectives on Participation in Education of Medical Students**	
Would become active in the training of medical students in Siegen	83% yes 17% no
One-day observation	80% yes 20% no
Two-week rotation	74% yes 26% no
Clinical elective	68% yes 32% no
Practical Year	58% yes 42% no
Lectures at a university	34% yes 66% no
Participation in research projects	68% yes 32% no
Recruitment of patients in practice	57% yes 43% no
**Qualification of Nonmedical Staff**	
Practice nurse	39% yes 61% no
Number of practice nurses	77% 1 16% 2 7% 4
Staff member currently doing the practice nurse curriculum	11% yes 89% no
Number of staff members currently doing the practice nurse curriculum	56% 1 44% 2
Staff member planning to do the curriculum	35% yes 65% no

**Table 2:** Contribution of classification variables to cluster formation indicated by effect size η^2^.

Variable	η^2^
Ever had a resident physician?	0.042
age_dicho	0.082
practice years_dicho	0.117
MBI:	
exhaustion	0.338
depersonalisation	0.412
personal accomplishment	0.126
Work Satisfaction Questionnaire:	
patient care	0.374
burden	0.125
income-prestige	0.067
personal rewards	0.350
professional relations	0.277

**Table 3:** Descriptive statistics of classification variables in Clusters 1 and 2, and effect sizes of differences between the two clusters.

Variable	Cluster 1 (*n* = 43)	Cluster 2 (*n* = 42)	Effect Size
Ever had a resident physician?	Yes: 51%	Yes: 31%	Cramér V: 0.21
age_dicho	>Median: 64%	>Median: 36%	Cramér V: 0.29
practice years_dicho	>Median: 68%	>Median: 33%	Cramér V: 0.34
MBI:			
exhaustion	10.4 (6.0)	20.7 (8.2)	d = 1.41
depersonalisation	1.6 (1.7) Huber M: 1.5	7.4 (4.6) Huber M: 6.9	d = 1.65
personal accomplishment	39.7 (5.7)	35.6 (5.1)	d = 0.75
Work Satisfaction Questionnaire:			
patient care	23.9 (2.9)	19.4 (3.0)	d = 1.53
burden	18.0 (4.5)	14.8 (3.9)	d = 0.75
income-prestige	16.1 (2.5)	14.7 (2.7)	d = 0.53
personal rewards	18.5 (1.9)	15.4 (2.3)	d = 1.45
professional relations	12.3 (1.4)	10.3 (1.7)	d = 1.22

Median of practice years = 18, Median of age = 54.
